# Association between single nucleotides polymorphisms in the *DR2D*, *ANKK1*, *COMT*, and *5-HTT* genes with temporomandibular disorder

**DOI:** 10.1590/1678-7757-2025-0258

**Published:** 2025-10-20

**Authors:** Otavio Augusto POZZA, Fernanda Mara de Paiva BERTOLI, Franciele TOPOLSKI, Paula Porto SPADA, Marilisa Carneiro Leão GABARDO, Juliana Feltrin de SOUZA, Erika Calvano KÜCHLER, João Armando BRANCHER

**Affiliations:** 1 Universidade Positivo Faculdade de Ciências da Saúde Programa de Pós-graduação em Odontologia Curitiba Paraná Brasil Universidade Positivo, Faculdade de Ciências da Saúde, Programa de Pós-graduação em Odontologia, Curitiba, Paraná, Brasil.; 2 Universidade Federal do Paraná Departamento de Estomatologia Curitiba Paraná Brasil Universidade Federal do Paraná, Departamento de Estomatologia, Curitiba, Paraná, Brasil.; 3 University Hospital Bonn Department of Orthodontics Medical Faculty Bonn Germany University Hospital Bonn, Department of Orthodontics, Medical Faculty, Bonn, Germany.; 4 Universidade Federal do Paraná Centro Politécnico Programa de Pós-graduação em Biologia Molecular e Celular Curitiba Paraná Brazil Universidade Federal do Paraná, Centro Politécnico, Programa de Pós-graduação em Biologia Molecular e Celular, Curitiba, Paraná, Brazil.

**Keywords:** Genetic polymorphisms, Orofacial pain, Temporomandibular joint disorders, Serotonin, Dopamine

## Abstract

**Background:**

Temporomandibular disorders (TMDs) comprise a heterogeneous group of musculoskeletal conditions affecting the temporomandibular joint, masticatory muscles, and associated structures. Growing evidence highlights the role of genetic predisposition as a significant contributor to TMD.

**Aim:**

This study aimed to investigate genetic aspects involved in TMDs etiology.

**Methodology:**

A cross-sectional study was conducted with 249 adolescents, of whom 149 were affected by TMD. Genomic DNA was extracted from buccal cells, and single nucleotide polymorphisms (SNPs) in *DRD2* (rs6275 and rs6276), *ANKK1* (rs1800497), *COMT* (rs6269 and rs4818), and *5-HTT* genes (rs3813034 and 1042173) were analyzed. Allelic and genotypic distribution, haplotype, and diplotype analysis were performed using PLINK software version 1.06. Multifactor dimensionality reduction (MDR) was applied to identify SNP-SNP interactions and generate an interaction graph.

**Results:**

In total, three possible single-locus allele combinations were obtained for haplotype and diplotype analyses (rs6275|rs6276 in *DRD2*, rs6269|rs4818 in *COMT* and rs3813034|rs1042173 in *5-HTT*), but no associations with TMD were observed (p>0.05). However, MDR analysis for gene-gene interactions revealed a synergistic relationship between rs6275 (*DRD2*), rs6269 (*COMT*), and rs1042173 (*5-HTT*) that predisposes to TMD (p=0.050).

**Conclusion:**

MDR analysis suggests a possible synergistic interaction among SNPs in the *DRD2*, *COMT*, and *5-HTT* genes that may contribute to TMD susceptibility in adolescents.

## Graphical abstract

**Figure:**
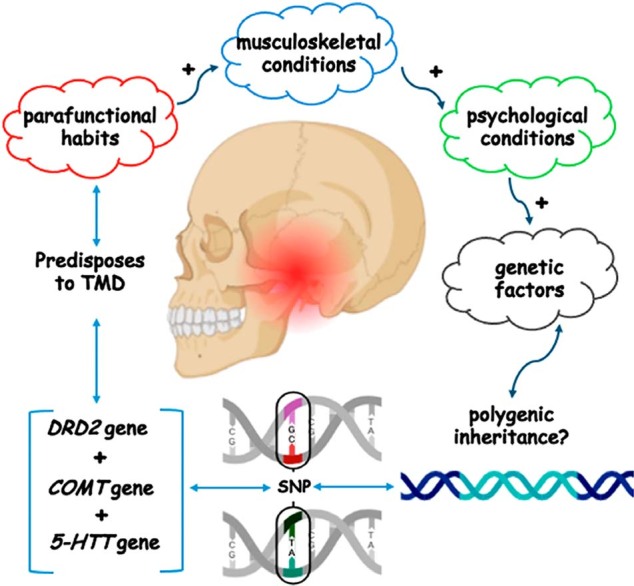

## Introduction

The term temporomandibular disorder (TMD) encompasses a group of musculoskeletal and neuromuscular conditions involving pain and/or dysfunction in the temporomandibular joint (TMJ), masticatory muscles, and related tissues.^1,2^ TMD is frequently associated with parafunctional habits, muscular dysfunction, and psychological conditions such as stress, depression, and anxiety,^3^ and is characterized by restricted mouth opening, mandibular movement deviations, and joint noises.^4^ TMD symptoms fluctuate from early childhood to adulthood,^5^ likely because they are associated with individual developmental stages.^6^In children and adolescents, signs and symptoms of TMD vary widely,^7,8^ and pain in the TMJ region, tenderness to palpation, muscle fatigue, joint sounds, and limited mandibular movement are frequently observed.^9^ In adults, TMD signs are more common in women than men and typically appear from the third decade of life.^10^

TMD has a multifactorial etiology with initiating,^11,12^predisposing,^13^ and perpetuating factors.^14^ Initiating factors trigger the onset of the pathology, predisposing factors increase the individual’s risk of developing it, and perpetuating factors interfere with recovery or contribute to the progression of the condition. In children, adolescents, and adults, TMD limits, and even incapacitate, daily activities. Thus, correct management must integrate individual physical and psychological processes.^15^

In recent years, our research group has investigated the prevalence of TMD in a population of adolescents in Curitiba, Brazil. Nearly 1,000 adolescents were examined, and the prevalence of TMD symptoms was 36.9%.^16^ Notably, high or moderate anxiety levels were found in 82.6% of adolescents, and those with high anxiety levels were 3.25 times more likely to show TMD symptoms. Since TMD was associated with anxiety in this sample, the next step was to investigate the association between TMD symptoms and single nucleotide polymorphisms (SNPs) in anxiety-related genes. To this end, we selected candidate genes that regulate endogenous mechanisms of pain, stress, and anxiety as follows: *Dopamine Receptors (DRD2), Ankyrin Repeat and Kinase Domain Containing 1 (ANKK1), Catechol-O-Methyl transferase (COMT),* and *Serotonin Transporter gene (5-HTT),* and to investigate their association with TMD in that population.

At that time, our findings indicated that SNPs in *5-HTT* were associated with painful TMD (arthralgia and myofascial pain); SNPs in *COMT* were significantly associated with myofascial pain and very close to being related to painful TMD and disc displacement.^17^ Moreover, the SNP rs6275 in *DRD2* was associated with disc displacement.^18^ In other words, most of the SNPs studied were individually associated with specific TMD symptoms.

**Figure 1:**
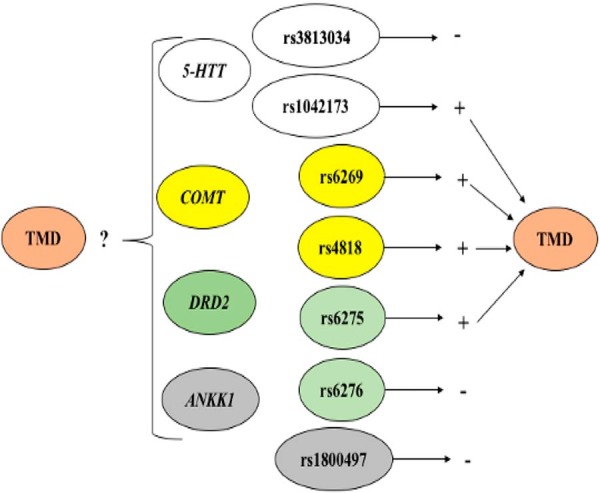
Framework of our study hypothesis. Single nucleotide polymorphisms in *COMT*, *5-HTT*17, and *DRD2*18 were individually associated with TMD (+). Is it possible that a complex interplay between these SNPs and genes contributed to the same outcome?

Based on this evidence, we hypothesized that a complex interplay among these SNPs and genes could increase the risk of TMD. To test this hypothesis, this case-control study aimed to evaluate haplotype- and diplotype-based associations in *DRD2* (rs6275, rs6276), *ANKK1* (rs1800497), *COMT* (rs6269, rs4818), and *5-HTT* (rs3813034 and rs1042173), as well as in gene-gene interactions by multifactor dimensionality reduction analyses in TMD in a population of Brazilian adolescents (Figure 1).

## Methodology

### Ethical approval, study design, and sampling

This cross-sectional study was approved by the Research Ethics Committee of the Federal University of Paraná (Process no. 2.006.086). Participants received written information about the study, and signed consent was obtained from a parent or legal guardian for all adolescents. The study followed the Declaration of Helsinki guidelines and was conducted according to the principles for reporting genetic association studies as defined by the STrengthening the REporting of Genetic Association Studies (STREGA) recommendations.^19^This study used a random sample selected from a previous cross-sectional survey by Bertoli, et al.^20^(2018) which included 934 adolescents of both sexes aged 10-14 years. Based on a previously reported TMD prevalence of 25% in adolescents,^17^ a subset of 249 adolescents was selected using a random number generator (www.randomizer.com), leading to 149 adolescents with a TMD diagnosis (TMD-affected) and 100 unaffected adolescents.

### Determination of the TMD phenotype

TMD phenotype was determined in two stages: first, the presence of self-reported TMD symptoms (myofascial pain, arthralgia, disc displacement, and painful TMD) was assessed using a validated Brazilian Portuguese version of the self-report questionnaire from the American Academy of Orofacial Pain.^21^ Adolescents with at least one positive answer were then clinically examined following the Research Diagnostic Criteria for Temporomandibular Disorders (RDC/TMD), which included muscle diagnosis encompassing myofascial pain, with or without limited opening; disc displacements, with or without reduction; and arthralgia, osteoarthrosis, and osteoarthritis.^22^ The presence of at least one of these signs or symptoms during clinical examination was considered to classify the adolescent as TMD affected. The sequence of clinical examination followed Bertoli, et al.^16^ (2028). The control group consisted of adolescents randomly selected without a TMD diagnosis.

### DNA samples and genotyping

Genomic DNA for molecular analysis was extracted from buccal cells based on the method described by Küchler, et al.^23^ (2012). The SNPs were selected from the International HapMap Project (www.hapmap.org), an international initiative to develop a map with patterns of DNA sequence variations. In this database, information about the SNPs in the genes of interest is available. The characteristics of the studied SNPs, including minor allele frequency (MAF) and base change, are as follows: two SNPs of the *DRD2* gene (rs6275, 0.473, A/G, and rs6276, 0.466, C/T), one SNP of the *ANKK1* gene (rs1800497, 0.325, G/A), two SNPs of the *COMT* gene (rs6269, 0.356, A/G, and rs4818, 0.296, C/G/T) and two SNPs of the *5-HTT* gene (rs3813034, 0.483, A/C, and rs1042173, 0.485, G/T) (retrieved from the NCBI database: ncbi.nlm.nih.gov).

### Statistical analysis

Chi-square test was applied to analyze the Hardy-Weinberg Equilibrium and to compare allele and genotype frequencies. Fisher’s exact test was used to compare haplotype frequencies with PLINK version 1.06 (https://zzz.bwh.harvard.edu/plink/ld.shtml). Poisson regression was performed for multivariate analysis adjusted by sex and age, and Prevalence Ratio (PR) with 95% Confidence Interval (95% CI) was obtained. IBM SPSS version 25.0 (IBM Corp., Armonk, USA) was used for these analyses, and values of p<0.05 indicated statistical significance.

Multifactor dimensionality reduction (MDR) adjusted for sex and age as co-variates was applied to identify SNP-SNP interactions. Two software programs, MDR 3.0.2^24^ and MDR Permutation Testing Module 1.0 beta 2^25^ (available at sourceforge.net/projects/mdr/files), were used to perform 10-fold cross-validation consistency (CVC), testing balanced accuracy (TBA), and run a 1000 permutation test to determine the statistical significance of the models. Models with a CVC of 9/10 or 10/10, TBA greater than 0.55, and p≤0.05 were considered the best models. Entropy values were estimated according to Jakulin and Bratko,^26^ and based on these values MDR created an interaction graph. Bonferroni correction was applied to control multiple comparisons within each set of analyses by multiplying the p-value by the number of tests in that group. Adjusted p-values greater than 1.000 were reported as 1.000, and only corrected p-values below 0.05 were considered significant.^27^

## Results

A total of 249 adolescents participated in this study. 139 (55.8%) were female, and 110 (44.2%) were male. In the TMD-affected group, the sample was composed of 149 (59.9%) adolescents. Among them, 92 females (61.7%) and 57 (38.3%) males. It is important to emphasize that TMD prevalence was significantly higher in females, representing a 1.61-fold greater prevalence than in males (p=0.022). Table 1 shows the allelic and genotypic frequencies in the TMD-affected and unaffected groups. No association was observed between TMD and the studied SNPs in the *ANKK1, COMT*, and *5-HTT* genes in either allelic or genotypic models (p>0.05).

**Table 1:** Allelic and genotypic frequencies in TMD-affected and unaffected groups.

Gene	SNP	Minor allele	Frequency in controls	Frequency in TMD cases	P#
*DR2D*	rs6275	C	0.3474	0.3926	0.323
	rs6276	A	0.3646	0.4046	0.387
*ANKK1*	rs1800497	C	0.2121	0.2263	0.714
*COMT*	rs6269	G	0.3073	0.3182	0.804
	rs4818	G	0.3258	0.3417	0.734
*5HTT*	rs3813034	C	0.4531	0.4228	0.516
	rs1042173	C	0.4694	0.4234	0.321

Note: # Chi-square test was performed

To investigate SNP × SNP interaction, haplotype analysis was performed, and three possible combinations of single-locus alleles were obtained: rs6275|rs6276 in *DRD2*, rs6269|rs4818 in *COMT,* and rs3813034|rs1042173 in *5-HTT*. None were associated with TMD (p>0.05) (Table 2). Diplotype analysis was used to investigate the specific variants of all possible combinations of single-locus genotypes in the studied genes. None of the diplotype combinations showed an increased probability of TMD (p>0.05) (Table 3).

**Table 2:** Haplotype analysis for *DRD2*, *COMT* and *5-HTT* genes.

Gene	SNPs	Haplotype	Frequency in controls	Frequency in TMD cases	P-value#
*DR2D*	rs6275|rs6276	C-A	0.3481	0.3871	0.408
		T-A	0.0110	0.0201	0.461
		T-G	0.6409	0.5927	0.312
*COMT*	rs6269|rs4818	G-G	0.2496	0.2704	0.642
		A-G	0.0680	0.0659	0.934
		G-C	0.0386	0.0482	0.644
		A-C	0.6437	0.6155	0.564
*5-HTT*	rs3813034|rs1042173	C-C	0.4492	0.4183	0.513
		A-C	0.0107	0.0152	0.680
		A-A	0.5401	0.5665	0.578

Note: * PLINK compare the frequencies between expected number of haplotypes by fisher test.

**Table 3:** Diplotype analysis using Poisson Regression adjusted for sex and age.

Genes	SNPs	Reference Diplotype	Diplotypes	PR (95% CI)	p-value
*DR2D*	rs6275 and rs6276	TT + GG	TC + GA	1.14 (0.71 – 1.83)	0.580
			CC + AA	1.25 (0.69 – 2.28)	0.452
*COMT*	rs6269 and rs4818	AA + CC	AG + CG	1.11 (0.68 – 1.80)	0.664
			GG + GG	1.09 (0.53 – 2.24)	0.804
*5-HTT*	rs3813034 and rs1042173	AA + AA	AC + AC	1.25 (0.77 – 2.02)	0.363
			CC + CC	0.85 (0.42 – 1.75)	0.674

Note: PR means Prevalence Ratio. 95% CI means 95% Confidence Interval.

**Figure 2:**
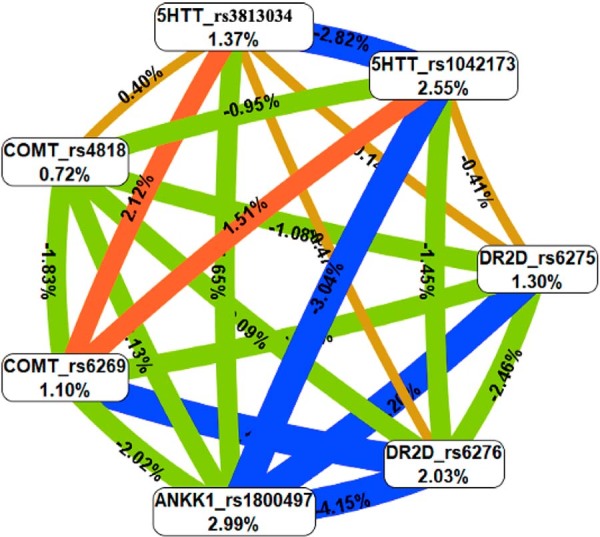
Entropy graph. This graph shows the entropy value of each SNP individually, indicated by percentages inside the nodes. The percentages on the lines indicate the entropy values resulting from the combination of SNPs. The different colors of the lines indicate the type of entropy, such as red and orange for synergic entropy and blue and green for redundancy entropy. The combination of rs6275 (*DRD2*), rs6269 (*COMT*), and rs1042173 (*5-HTT*) reflects a non-robust tendency to predispose to TMD.

Next, between-gene multifactor dimensionality reduction (MDR) analysis was performed to test whether SNP × SNP combinations and gene-gene interactions contributed to increased risk of TMD. Among the results, the best combination and interaction, with the highest balanced accuracy, was SNPs rs6275 in *DRD2* gene, rs6269 in *COMT* gene, and rs1042173 in *5-HTT* gene. Nonetheless, this combination was not statistically significant (p=0.050) and reflected only a tendency toward a synergistic relationship between these SNPs and genes in increasing TMD risk (Table 4, Figure 2). In addition, Bonferroni correction applied to this combination revealed a non-robust causal relationship (p=0.357).

**Table 4:** Summary of MDR analysis results for TMD.

Locus Number	Best Combination	TBA#	CV*	P-valueǂ
2	rs6269 (*COMT*), rs1042173 (*5-HTT*)	0.5707	07/10	0.259
3	rs6275 (*DRD2*), rs6269 (*COMT*), rs1042173 (*5-HTT*)	0.6042	10/10	**0.050**
4	rs6275 (*DRD2*), rs1800497 (*ANKK1*), rs6269 (*COMT*), rs1042173 (*5-HTT*)	0.5316	03/10	0.695
5	rs6275 (*DRD2*), rs6276 (*DRD2*), rs1800497 (*ANKK1*), rs6269 (*COMT*), rs1042173 (*5-HTT*)	0.5501	10/10	0.117
6	rs6275 (*DRD2*), rs6276 (*DRD2*), rs1800497 (*ANKK1*), rs4818 (*COMT*), rs38133034 (*5-HTT*), rs1042173 (*5-HTT*)	0.5389	05/10	0.595
7	rs6275 (*DRD2*), rs6276 (*DRD2*), rs1800497 (*ANKK1*), rs6269 (*COMT*), rs4818 (*COMT*), rs3813034 (*5-HTT*), rs1042173 (*5-HTT*)	0.5274	04/10	0.742

Notes: # Testing Balanced Accuracy. * Cross-validation consistency. ǂ P-values were based on 1000 permutations test.

## Discussion

It is not new that TMD is a complex condition involving environmental and intrinsic factors, including genetic background. It is also commonly accepted that genetic variants may predispose to increased risk for TMD.^28^ Nonetheless, can different genes interact to cause TMD? This is the crucial point of this cross-sectional study. To answer this question, we used powerful tools for genetic analysis, including haplotype, diplotype-based association, and multifactor dimensionality reduction analyses. DNA samples from 249 Brazilian adolescents, most of them affected by TMD, were analyzed in *DRD2* (rs6275, rs6276), *ANKK1* (rs1800497), *COMT* (rs6269, rs4818), and *5-HTT* (rs3813034 and rs1042173). Although MDR analysis suggested a possible synergistic interaction between rs6275, rs6269, and rs1042173 in TMD predisposition, none of the six gene-gene combinations showed significant positive interactions or additive effects for TMD risk.

Although a disorder, research groups worldwide have revealed that TMD follows the biopsychosocial disease model, in which multiple factors such as anxiety,^3,20^ depression,^29^ physical, psychological, and socio-demographic aspects,^30^ hormones,^31,32^ and genetic background,^17,18,33-35^act as triggers. Many studies report a higher prevalence of TMD in females. One of the hypotheses raised for this association is that estrogen levels fluctuate drastically during the menstrual cycle, and excessive levels, particularly in the early follicular phase, may potentiate TMD signs and symptoms.^36^ In addition, estrogens play a molecular signaling role in the temporomandibular joint^37^ via estrogen receptors encoded by Estrogens Receptors alpha and beta (*ESR*α and *ESR*β) genes.^38^ In this sense, Berger, et al.^31^ (2015) previously highlighted the importance of estrogen level assessment in studies on TMD.

Our study reinforced that TMD is significantly higher in females, perhaps due to stronger estrogen modulation in these female adolescents. Unfortunately, we did not address pubertal stage or estrogen levels in the evaluated population. Our sociodemographic questionnaire included a question about menarche, and only a small sample of girls had reported it. Another point to note is that *ESR*α and *ESR*β are also anxiety-related genes, as estrogen impacts the limbic system by modulating behavior, emotional status, and pain perception,^39^ all of which are factors related to TMD. Therefore, *ESR* genes should have been included in the MDR analyses. The absence of estrogen data and of *ESR* genes are limitations of our study.

In 2018, a review listed 73 genes that were somehow linked to TMD, including genes expressed in skeletal muscle, genes involved in aggrecan cleavage, collagen pathways, bone metabolism, and catecholamine, serotonin, and dopamine metabolism, among others. Most studies investigated specific SNPs in TMD-affected individuals through allelic or genotypic analyses.^28^ Currently, several functional SNPs are available among candidate genes related to TMD.^33-35^Our group also identified some SNPs associated with TMD, including an association between the *COL2A1* gene and temporomandibular joint disc displacement^40^and between SNPs in 5-*HTT, COMT,* and *DRD2* genes with TMD symptoms.^17,18^These three genes were target genes because their association with episodes of anxiety and TMD had already been found, but all analyses performed were based only on the contribution of each SNP or gene separately to TMD outcomes.

Investigations based only on alleles or genotypes can be complex and may not effectively detect associations between SNPs and the studied outcome.^1^ Therefore, tools that explore SNP-SNP interactions, as well as haplotypes and diplotypes, are essential for addressing this gap. These tools can reflect contributions from multiple loci and help predict interactions that may negatively or positively impact a given disease or condition.^42^ Since haplotype and diplotype analyses are more accurate due to the preservation of additional information,^41^ we decided to apply them to the studied SNPs to test the hypothesis that an association between SNPs and genes could reflect contributions from multiple loci to TMD outcome. Three possible single-locus alleles and single-locus genotypes were obtained, none of which showed association with TMD. This lack of association can be explained in different ways: (i) environmental modifiers outweigh genetic effects at these loci; (ii) molecular regulatory mechanisms not captured by haplotypes/diplotypes; (iii) population heterogeneity; or (iv) in fact, TMD depends on single-variant effects of the studied SNPs rather than haplotype or diplotype blocks.

Lastly, MDR analysis was performed. This analysis combines testing balanced accuracy, cross-validation consistency, and dimensionality reduction techniques to reduce the dimensionality of multilocus genotypes and detect gene-gene interaction, which increases the accuracy of estimates of the prediction error rate even in small population samples.^43^ Through the MDR technique, the genotypic analyses enable pooling individuals into high-risk and low-risk groups, effectively reducing genotype dimensionality to one dimension.^44^In addition, MDR cross-validation consistency enables the division of data into ten equal parts, increasing the model’s predictive capacity via independent data analysis and identifying significant high-order interactions among different polymorphisms and genes that other statistical tests fail to capture. A clear example of how this combined analysis can be helpful is the study by Ritchie, et al.^44^ (2001), one of the first to use MDR analysis. That study identified a statistically significant high-order interaction among four polymorphisms from three different estrogen-metabolism genes that, when analyzed separately, were not associated with the cancer outcome.

Regarding MDR results, our study suggests a specific interaction between *DRD2, COMT,* and *5-HTT* genes that may predict TMD, although the evidence is non-robust. The combination of rs6275, rs6269, and rs1042173 was the model with the lowest prediction error, highest cross-validation consistency, and best accuracy in MDR, suggesting a synergistic action in predisposing to TMD; however, this finding was not statistically supported (p=0.050). Interestingly, these same SNPs were individually associated with TMD in previous studies,^17,18^ which makes the possibility of synergism between them to increase the risk of TMD plausible, a hypothesis that was not confirmed. These null findings suggest that TMD risk is not driven by strong epistasis among the SNPs and genes studied. In our study, to increase the power of the MDR analysis, our research protocol considered two essential points: a) the number of study participants, and b) the minor allele frequency of the selected SNPs. Regarding our sample, 249 adolescents were included, which statistically represents the age of the target population, including 149 affected by TMD. Concerning SNP selection, it is important to emphasize that selecting SNPs with low minor allele frequency means rare SNPs and lower statistical power,^45^ i.e., the probability of not observing the association using rare SNPs increases substantially. In this study, we established a protocol for selecting SNPs with allele frequency close to 0.5, which reduces distortions and provides the best prediction model for most phenotypes.

Finally, in previous studies concerning these specific SNPs, we described their contributions to TMD susceptibility. In this study, a new approach was employed to investigate gene-gene interaction within these loci, and the results provide evidence that they influence the TMD outcome via independent biological mechanisms, without potential epistatic effects. In this sense, this work contributes to TMD genetics research by evaluating polygenic interaction, a crucial step toward understanding the condition’s complex etiology.

## Conclusion

MDR analysis suggests a possible, but non-robust, synergistic interaction among SNPs in *DRD2, COMT*, and *5-HTT* genes that may contribute to TMD susceptibility in adolescents.
